# A Rare Case of Disseminated Histoplasmosis With Hemophagocytic Lymphohistiocytosis Mimicking a Flare of Systemic Lupus Erythematosus in a Middle-Aged Man: A Case Report

**DOI:** 10.7759/cureus.46068

**Published:** 2023-09-27

**Authors:** Allen C Omo-Ogboi, Sara Shirai, Asad Ur Rehman, Joyce O Ederhion, Maximilian Buja

**Affiliations:** 1 Department of Pathology and Laboratory Medicine, McGovern Medical School, University of Texas Health Science Center at Houston, Houston, USA; 2 Department of Neuroscience and Immunology, University of Roehampton, London, GBR

**Keywords:** organomegaly, immunosuppressive, disseminated histoplasmosis, hemophagocytic lymphohistiocytosis, systemic lupus erythematosus

## Abstract

Disseminated histoplasmosis is a progressive granulomatous disease caused by *Histoplasma capsulatum*, which is an intracellular dimorphic fungus endemic to the Ohio and Mississippi River valleys in the United States. It is usually thought to be due to the failure of the activation of the T-cell-mediated immune response. Hemophagocytic lymphohistiocytosis (HLH) is a rare but potentially fatal condition, in which histiocytes and lymphocytes build up in and damage organs and other blood cells. We present a 37-year-old man with a past medical history of systemic lupus erythematosus (SLE) complicated by lupus nephritis on immunosuppressive therapy who presented to the emergency department with hypotension and was admitted for acute kidney injury. Prior to the presentation, he had persistent fever, myalgias, cough, mild shortness of breath, and back pain. Computed tomography (CT) chest shows “eggshell” calcification; microbiology evaluation of peripheral blood smear revealed intracellular organism, morphologically consistent with *H. capsulatum*; and urine histoplasmosis antigen test confirmed the diagnosis of histoplasmosis. HLH diagnosis was made clinically after “clinical and testing criteria” were evaluated. Despite further management, he developed coagulopathy and sepsis, which led to his death. At autopsy, we found organomegaly of the liver, spleen, and kidneys. Microscopically, these enlarged organs show old fibrotic granulomas and granulomatous inflammation with suspected fungal organisms. Gomori's methenamine silver special stain confirmed these fungal organisms to be consistent with *Histoplasma *species (3-5 micron budding yeasts). This case highlights that physicians should be aware of the diagnostic challenge that disseminated histoplasmosis with HLH could pose in a patient with SLE, especially in patients on immunosuppression. Failure to recognize the infection promptly could lead to grievous complications and possibly death.

## Introduction

Disseminated histoplasmosis is a progressive granulomatous disease caused by *Histoplasma capsulatum*, which is an intracellular dimorphic fungus endemic to the Ohio and Mississippi River valleys in the United States [1]. The progressive spread of the infection to organs is thought to be due to the failure of the activation of the T-cell-mediated immune response, which is typically completed within two weeks of exposure to this organism [2]. Histoplasmosis can spread to every organ system during dissemination [3]. Disseminated histoplasmosis is more commonly seen in immunocompromised individuals with impaired cell-mediated immune responses. Acute disseminated histoplasmosis is characterized by high fatality rates and abrupt onset of symptoms, including fever, malaise, hepatosplenomegaly, lymphadenopathy, anemia, leukopenia, and thrombocytopenia. Skin lesions and pancytopenia are more commonly seen in immunocompromised compared to immunocompetent individuals [4]. Diagnosing disseminated histoplasmosis with hemophagocytic lymphohistiocytosis (HLH) in a patient with systemic lupus erythematosus (SLE) is diagnostically challenging due to the overlapping presentation. Hence, we are reporting this case to alert physicians to keep a high index of suspicion in such presentations.

## Case presentation

The decedent was a 37-year-old male with a past medical history of type 1 diabetes mellitus, hyperlipidemia, hypertension, and SLE complicated by lupus nephritis (ISN/RPS class IV) and grade II diastolic congestive heart failure (on prednisone, tacrolimus, mycophenolate mofetil) who presented to the emergency department (ED) from same day clinic for hypotension (85/40). Prior to the presentation, he reportedly had two weeks of constitutional symptoms (persistent fever, myalgias, and back/neck pain). These symptoms were associated with mild shortness of breath, rare cough, weakness, and several episodes of non-bloody diarrhea. He was admitted for acute kidney injury.

**Figure 1:**
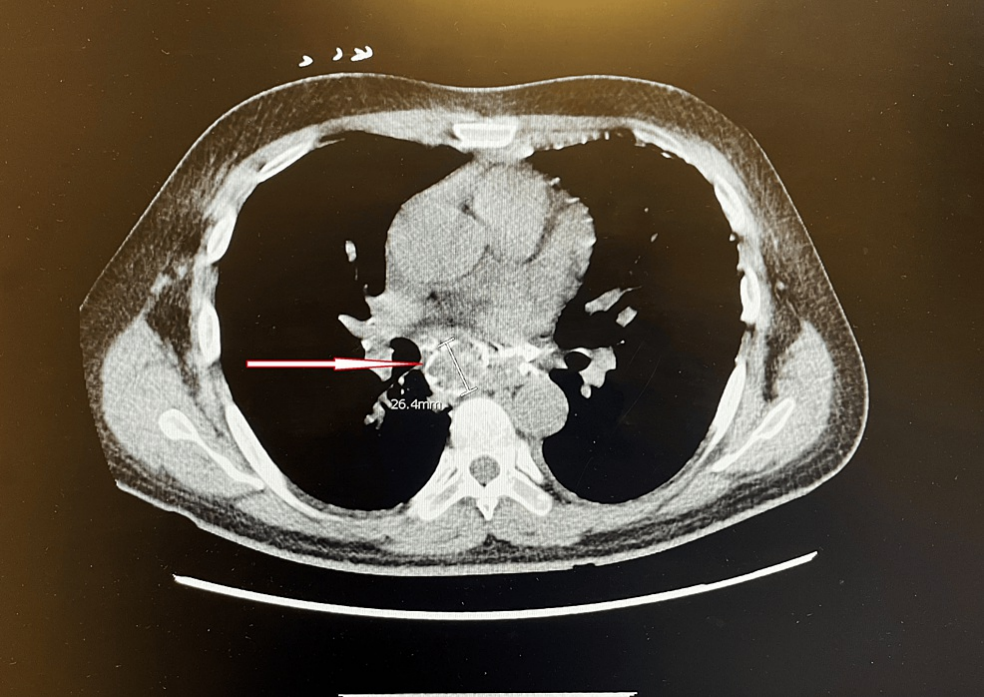
Axial computed tomography (CT) scan showing eggshell calcification (red arrow).

He developed a fever (highest 103.2 F) during the admission, and a diagnosis of HLH was made clinically after the “clinical and testing criteria” were evaluated by the clinical team. Computed tomography (CT) chest showed "eggshell type" calcification (Figure 1) and partially calcified intrathoracic lymphadenopathy and upper lobe predominant subpleural/perilymphatic micronodules. Microbiology evaluation of peripheral blood smear revealed an intracellular organism, morphologically consistent with *Histoplasma*, and urine histoplasmosis antigen test confirmed the diagnosis of histoplasmosis. A transthoracic echocardiogram showed possible vegetation of the aortic valve. The hospital course was complicated by sepsis and anemia unresponsive to transfusion, and he continued to have fever, tachycardia, and tachypnea. About a week later in the morning, he became unresponsive, and CPR was performed; however, he was declared deceased shortly after.

An unrestricted autopsy of the decedent revealed multiple findings related to disseminated histoplasmosis infection and his underlying co-morbidities, SLE in particular. His heart was grossly notable for concentric left ventricular hypertrophy, as well as bicuspid aortic valve with a "false commissure." Bilateral lungs were noted to be diffusely firm and airless. Enlargement of bilateral kidneys (710 g, combined), spleen (470 g), and liver (1,950 g) with fatty liver change were also noted. The decedent had a malar rash and diffuse erythematous macules on the body surface.

**Figure 2:**
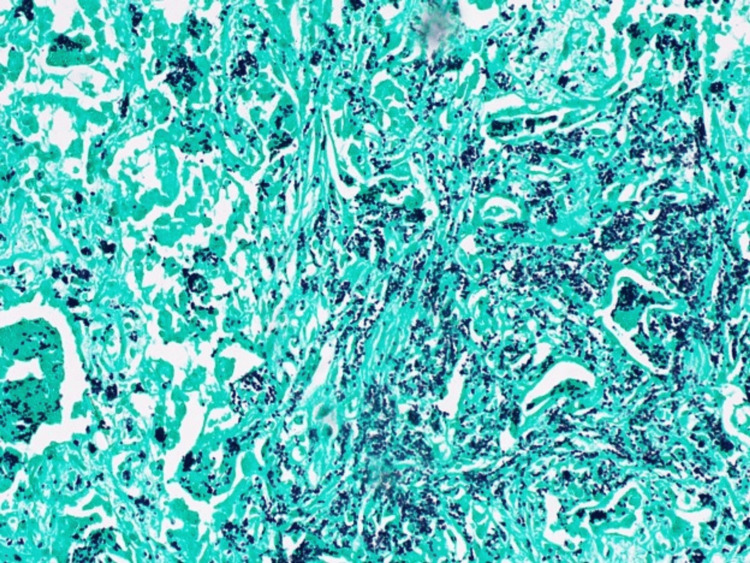
Lung lesion showing Histoplasma organisms on Grocott's methenamine silver stain diffusely infiltrating the lung (20x).

**Figure 3:**
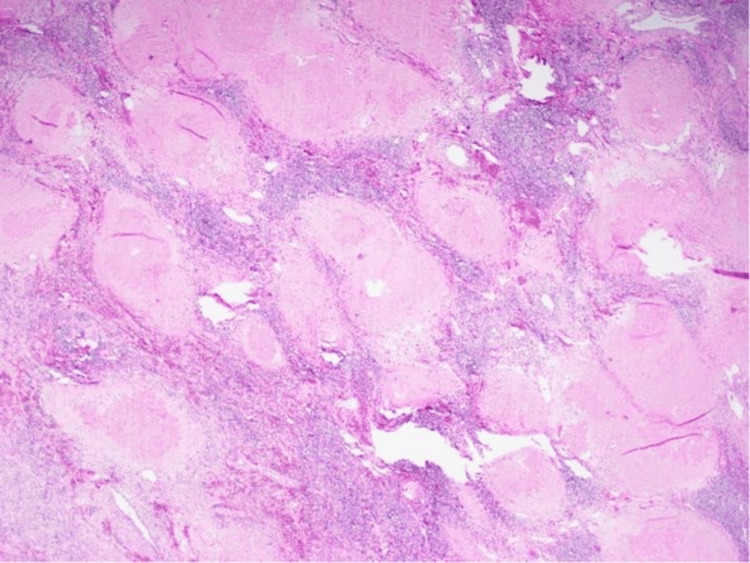
Hilar lymph node shows old fibrotic granulomas and granulomatous inflammation on hematoxylin and eosin stain (20x).

**Figure 4:**
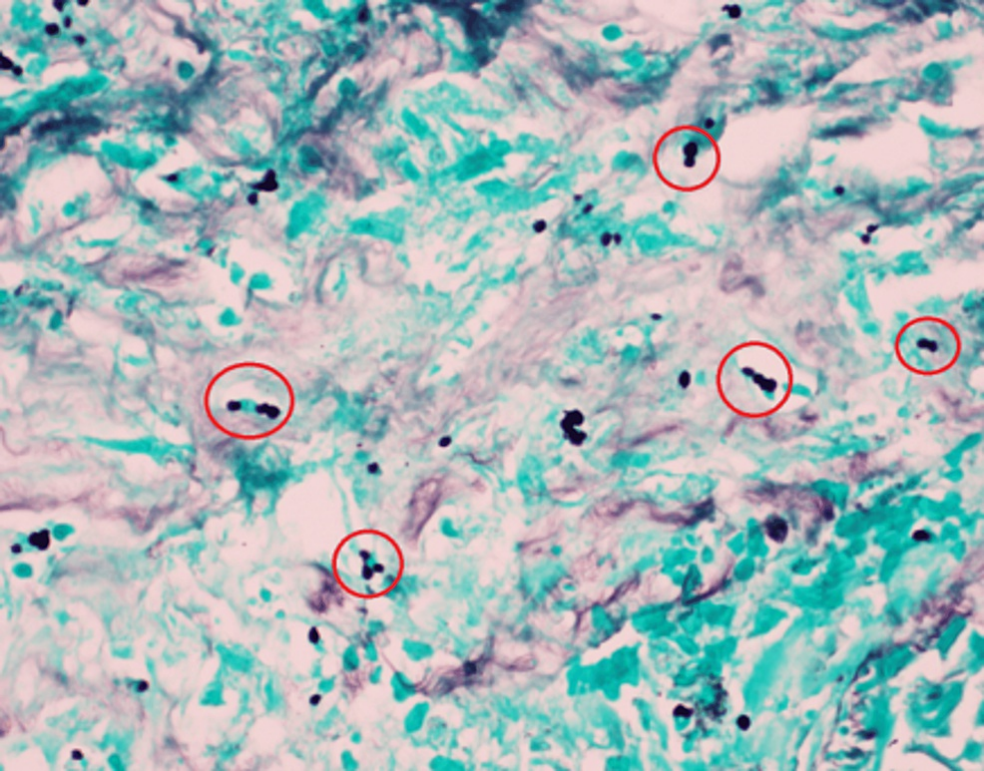
Higher magnification of (Figure 3) showing Histoplasma organisms identified in "red circles" on Grocott's methenamine silver stain (60x).

Microscopically, lungs show a lesion with a necrotic center surrounded by macrophages, encapsulating fibroblasts, fibrous connective tissue in the periphery, and scattered lymphocytes with organisms suggestive of *Histoplasma* species (3-5 micron budding yeasts). Gomori's methenamine silver special stain further confirmed these organisms as *Histoplasma *(Figure 2), given prior confirmation by the urine histoplasmosis antigen test. The hilar lymph node shows old fibrotic granulomas and granulomatous inflammation on hematoxylin and eosin stain (Figure 3), and higher magnification on Gomori's methenamine silver special stain showed *Histoplasma *organisms (Figure 4). The liver, spleen, bilateral kidneys, and other internal organs also had the *Histoplasma *organisms; hence, an assessment of disseminated histoplasmosis was made.

## Discussion

Histoplasmosis is caused by the thermally dimorphic fungus *H. capsulatum* and is the most prevalent endemic mycosis in the United States with endemic areas located in the Mississippi and Ohio River valleys [5,6]. It can be linked with exposure to birds and bat droppings. Two varieties of *H. capsulatum* are pathogenic to humans, *H. capsulatum *var. *capsulatum *and *H. capsulatum *var. *duboisii*, with the former predominant in North and Central America and the latter in Africa [3,5]. Histoplasmosis can present in several ways, from localized pulmonary involvement to life-threatening disseminated disease, as seen in our case [5]. Disseminated histoplasmosis has been described in patients who are immunocompromised, as seen in our case, as which the decedent has a history of SLE and was on multiple immunosuppressive therapies [5].

HLH is a fatal condition caused by an overactive immune state [5,7]. HLH can be primary or secondary to infections (mostly associated with Epstein-Barr virus, cytomegalovirus, parvovirus B19, and HIV), malignancy, and inherited immunodeficiency disorders [5,7]. HLH secondary to histoplasmosis is very rare, accounting for less than 1% of cases worldwide [5,8]. The mechanism of histoplasmosis-triggered HLH has not been fully explained. However, it is likely related to a T-cell-mediated immune response, macrophage activation, and cytokine storm to an underlying trigger [5,9]. Mortality with HLH-associated histoplasmosis has a fatality rate of 31% [5,10].

In our case, the decedent presented with persistent fever, myalgias, and back/neck pain, which can be found in both SLE flares due to his previous history and can present as a new presentation of HLH, as seen in this case. The history of SLE with multiple immunosuppressive therapy, which gave rise to disseminated histoplasmosis due to the immunosuppressive state and secondarily led to HLH. Performing radiologic and laboratory investigations further helps in differentiating these clinically identical entities; like in our case, CT chest showed "eggshell type" calcification and partially calcified intrathoracic lymphadenopathy, and upper lobe predominant subpleural/perilymphatic micronodules, hepatosplenomegaly, which was eventually confirmed to be disseminated histoplasmosis, secondarily linked to HLH. The autopsy findings and his clinical presentation represent his underlying SLE, ongoing HLH, as well as manifestation of disseminated histoplasmosis infection, including fever, firm and airless lungs, hepatosplenomegaly, diffuse erythematous macules, transfusion-refractory anemia, coagulopathy, and sepsis, which eventually led to his death.

## Conclusions

In conclusion, this case highlights that physicians should be aware of the diagnostic challenge that disseminated histoplasmosis with HLH could pose in a patient with SLE, especially in patients on immunosuppression. The clinical presentation could easily mimic a flare of SLE, which could affect treatment and overall prognosis. Failure to recognize the infection promptly could lead to grievous complications and possibly death.
